# Test-treatment RCTs are susceptible to bias: a review of the methodological quality of randomized trials that evaluate diagnostic tests

**DOI:** 10.1186/s12874-016-0287-z

**Published:** 2017-02-24

**Authors:** Lavinia Ferrante di Ruffano, Jacqueline Dinnes, Alice J. Sitch, Chris Hyde, Jonathan J. Deeks

**Affiliations:** 1 Biostatistics, Evidence Synthesis and Test Evaluation Research Group, Institute of Applied Health Research, University of Birmingham, Birmingham, B15 2TT UK; 2 PenTAG, Institute of Health Research, University of Exeter Medical School, Exeter, EX1 2LU UK

**Keywords:** RCT, Test-treatment, Test evaluation, Methodological quality, Diagnostic accuracy, Patient outcomes, Bias

## Abstract

**Background:**

There is a growing recognition for the need to expand our evidence base for the clinical effectiveness of diagnostic tests. Many international bodies are calling for diagnostic randomized controlled trials to provide the most rigorous evidence of impact to patient health. Although these so-called test-treatment RCTs are very challenging to undertake due to their methodological complexity, they have not been subjected to a systematic appraisal of their methodological quality. The extent to which these trials may be producing biased results therefore remains unknown. We set out to address this issue by conducting a methodological review of published test-treatment trials to determine how often they implement adequate methods to limit bias and safeguard the validity of results.

**Methods:**

We ascertained all test-treatment RCTs published 2004–2007, indexed in CENTRAL, including RCTs which randomized patients to diagnostic tests and measured patient outcomes after treatment. Tests used for screening, monitoring or prognosis were excluded. We assessed adequacy of sequence generation, allocation concealment and intention-to-treat, appropriateness of primary analyses, blinding and reporting of power calculations, and extracted study characteristics including the primary outcome.

**Results:**

One hundred three trials compared 105 control with 119 experimental interventions, and reported 150 primary outcomes. Randomization and allocation concealment were adequate in 57 and 37% of trials. Blinding was uncommon (patients 5%, clinicians 4%, outcome assessors 21%), as was an adequate intention-to-treat analysis (29%). Overall 101 of 103 trials (98%) were at risk of bias, as judged using standard Cochrane criteria.

**Conclusion:**

Test-treatment trials are particularly susceptible to attrition and inadequate primary analyses, lack of blinding and under-powering. These weaknesses pose much greater methodological and practical challenges to conducting reliable RCT evaluations of test-treatment strategies than standard treatment interventions. We suggest a cautious approach that first examines whether a test-treatment intervention can accommodate the methodological safeguards necessary to minimize bias, and highlight that test-treatment RCTs require different methods to ensure reliability than standard treatment trials.

Please see the companion paper to this article: http://bmcmedresmethodol.biomedcentral.com/articles/10.1186/s12874-016-0286-0.

## Background

Diagnostic tests are an essential component of the clinician’s armory for deciding how best to manage their patients. But how should clinicians identify the ‘best’ test to use for a given indication? In an ideal world, such decisions would be guided by large meta-analyses of rigorous clinical effectiveness studies that summarize how competing tests impact on downstream patient health. In reality, there is a serious paucity of this evidence for most diagnostic procedures in use today [1–7]. Acknowledging that diagnostic accuracy studies alone are insufficient to demonstrate the clinical utility of tests, international bodies are increasingly calling for randomized controlled trials to provide the most rigorous evidence of impact to patient health [8, 9]. By analogy to the study design hierarchies for evaluating treatments, RCTs are commonly stated to be the ‘gold standard’ design for evaluating the effectiveness of tests [7, 10–15].

**Fig. 1:**
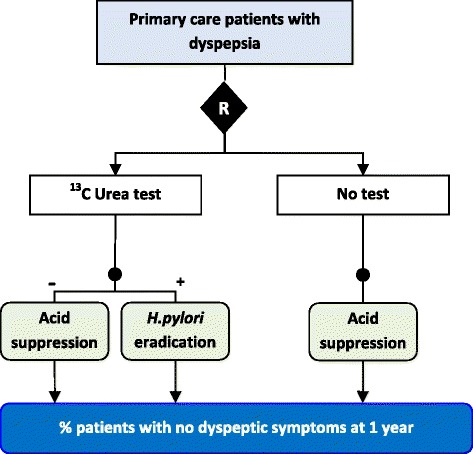
Example of a replacement test-treatment RCT. Patients randomized to the experimental arm receive a test for the presence of *Helicobacter pylori*, which is eradicated if found, while patients without bacterial infection are given proton pump inhibitors (acid suppression). Patients randomized to the control arm receive no test and are all given proton pump inhibitors (acid suppression), reflecting standard care. The outcome of the trial is eradication of dyspeptic symptoms at 12 months [16]

These ‘test-treatment’ RCTs randomise patients to undergo either a new test, or an existing test, measuring the downstream health response after patients have received subsequent treatment. Therefore when we seek to evaluate tests we must compare entire management pathways, called ‘test-treatment’ strategies, rather than single interventions. The MRC-CUBE trial, for example, evaluated whether testing dyspeptic patients for the bacterium *Helicobacter pylori* and treating those positive with eradication therapy, would effectively reduce their symptoms when compared to the standard approach of giving acid suppression to all dyspeptic patients [16] (Fig. 1). Test-treatment comparisons can take three general formats, depending on the role the new test will take within the existing strategy [17]. The MRC-CUBE trial describes a replacement comparison where the new test completely replaces the existing technique (in this case no testing), however RCTs can also measure the value of adding a new test either alongside the existing strategy (e.g. the RATPAC trial [18]), or earlier in the pathway, to select which patients will go on to receive the existing tests (e.g. the RELAPSE trial [19]).

Test-treatment RCTs have however attracted criticism [20]. Randomizing participants to testing strategies and measuring patient outcomes after treatment, test-treatment RCTs compare multi-staged interventions and face practical challenges in ensuring that they adequately control for bias. As with other complex interventions, the ability to blind could be compromised since test results must be interpreted by clinicians, and diagnoses recounted to their patients; it may therefore be impossible to eliminate performance and ascertainment bias [17, 21–23]. The need for patients to progress through multiple interventions (tests and treatments) could increase the proportion who drop-out, and since the quality and information patients receive differ according to the interventions used, these trials may also be susceptible to differential drop-out, placing them at increased risk of attrition bias [22]. In addition, sample sizes must be considerably larger in order to account for the probability that effects are only experienced in patients who receive different care as a result of their diagnoses; these trials may therefore risk being underpowered to detect patient health effects [1, 22].

These features of trial design have been empirically demonstrated to defend the RCT design against risks of considerable bias [24–26], yet the ability of test-treatment trials to implement methodological safeguards have not been systematically examined. Recognizing the increasing calls for RCTs to evaluate diagnostic tests, we undertook a review of published test-treatment trials to appraise the extent to which they are susceptible to the biases and challenges that are claimed to confront them. We compare our findings to similar reviews of pharmaceutical and complex intervention trials to evaluate whether observed inadequacies are due to the complex nature of test-treat interventions.

## Methods

The objective of this review was to describe the frequency with which test-treatment trials implement adequate methods to limit bias and safeguard the validity of results.

### Study sample

The Cochrane Central Register for Controlled Trials (CENTRAL) includes RCTs identified from MEDLINE and EMBASE, and records retrieved through hand-searching undertaken by Cochrane Review Groups and searching other sources. CENTRAL was searched (25th May 2009) for reports of test-treatment RCTs published between 2004 and 2007 (Table 1). Details of the search and selection process have been reported elsewhere [6, 27]. Briefly, eligible trials randomized patients between diagnostic testing strategies and measured at least one patient outcome occurring after treatment. Trials evaluating asymptomatic screening or monitoring tests were excluded, as were non-English language reports. Multiple reports of a single trial were assimilated through cross-referencing.

**Table 1:** Search strategy for test-treatment RCTs conducted in CENTRAL Issue 2 2009

Search strategy		Hits
#1	sensitiv^a^ or diagnose or diagnosis or diagnostic^a^ in Clinical Trials	70,052
#2	random^a^ in Clinical Trials	335,175
#3	“study design” next “rct” in Clinical Trials	150,275
#4	(#2 OR #3)	449,453
#5	(#1 AND #4)	50,419
#6	(#5), from 2004 to 2007	12,892

^a^denotes truncation of search term

### Data extraction

Trials were classified by journal type, clinical specialty, trial design, number of randomized groups, care setting and type of diagnostic comparison (triage, add-on, replacement) as defined by Bossuyt and colleagues [28]. Methodological items assessed internal validity and trial conduct according to: implementation of random sequence generation; allocation concealment; blinding of participants, care-givers and outcome assessors; definition and description of primary outcomes; numbers of cross-overs, drop-outs and other losses to follow-up; analytical approach including use of intention-to-test; and inclusion of sample size calculations. These items were identified from three validated, internationally accepted standards for the conduct and reporting of RCTs: the CONSORT checklist [29], the extension of the CONSORT statement for non-pharmacologic interventions [30] and the Cochrane Collaboration’s ‘Risk of Bias’ tool [31]. A standardized form was designed detailing the criteria used as given in Table 2.

**Table 2:** Definitions and criteria used to appraise the quality of trial methods and conduct

**1. Did methods of sequence generation adequately protect against selection bias?** *Clear description of method for allocating participants to study groups. Quality judged as* ***Adequate*** *,* ***Inadequate*** *or* ***Unclear*** *using criteria recommended by the Cochrane Collaboration* [31].	
**2. Did methods of allocation concealment adequately protect against selection bias?** *Clear description of method for preventing knowledge or prediction of group allocation amongst patients and care-providers. Quality judged as* ***Adequate***, ***Inadequate*** *or* ***Unclear*** *using criteria recommended by the Cochrane Collaboration* [31].	
**3. Were participants, care-providers and outcome assessors blinded to test-treatment interventions?** *Clear reports of whether participants, care-providers (those responsible for patient management) and outcome assessors were masked to the identity of tests used for decision-making, and a description of methods used to mask.*	
**4. Were primary outcomes comprehensively reported?** *Reports considered* ***adequate*** *with clear definition of the primary outcome and description of method and timing of measurement. When the primary outcome was not clearly defined, the outcome used in the power calculation, or if not the outcome stated in the primary study objective was considered as primary. The primary outcome was as ‘not defined’ in the absence of this information* [56]. *Outcomes were classified as patient based or process.* *Method of measurement considered* ***adequate*** *if a validated tool used, if non-validated but fully described tool used, or if rigorous criteria to assess outcome were provided (e.g. the operational definition of a target condition and test methods used to arrive at a diagnosis). Documentation considered* ***complete*** *when the time at which the primary assessment should be conducted was also made explicit.*	
**5. For each group, is it clear whether some participants did not receive the allocated intervention, were lost to follow-up, or were not analyzed?** *Clear and complete accounting of participant flow as detailed in CONSORT* [30], *including use of a CONSORT diagram. Reporting considered* ***adequate*** *if all five elements (Eligibility, Allocation, Receiving intervention, Followed-up, Analyzed) were reported for all study groups, and if these numbers agreed (e.g. if the number analyzed tallied with the numbers randomized and lost to follow up).* ***Analysis according to allocated group*** *–considered* ***inadequate*** *if patients not analyzed according to allocated study group, regardless of test(s) actually received.* ***Use of intention-to-treat*** *(ITT)–clear statement that ITT principle was used. Considered* ***adequate*** *if all study patients were analyzed as randomized, and analyses were complete.* ***Exclusions and missing data*** *–Clear description of numbers and reasons for missing data due to: missing outcome responses, exclusion of participants, and loss to follow-up; Description of methods used to deal with missing data* ***Complete analysis*** *–Analyses considered* ***complete*** *when no data were missing due to exclusions, missing responses or loss to follow-up for the primary outcome measured at the primary time-point. Magnitude of attrition calculated per group for each trial by comparing numbers randomized to numbers analyzed. Differential attrition considered as ≥5% and ≥20% difference between arms, following the approach advocated by the Centre for Evidence Based Medicine when judging the quality of comparative evidence of effectiveness* [57].	
**6. Was the primary analyses conducted appropriately?** ***Whole group analysis*** *–Primary outcomes measured in subgroups of the randomized population were considered* ***Inadequate*** * due to risk of selection bias* [58]. ***Inconsistency*** *–Use of different outcome assessment methods in each group considered* ***inadequate*** *unless the outcome was a measure of test performance (e.g. diagnostic yield or therapeutic yield).*	
**7. How did studies determine sample size?** *Reporting of a power calculation and outcome variable on which it was based, extraction of target sample size and comparison to achieved sample size.*	

This tool was piloted on five randomly selected test-treatment trials by three authors (JJD, CH, LFR) and modified to improve consistency. One reviewer undertook extraction and quality assessment of all trials (LFR) and a second reviewer (JaD) independently extracted and assessed a 64% convenience sample (the first 66 eligible trials). Disagreements were discussed to reach consensus judgements. If uncertainties remained after discussion, the data were checked by a third member of the team and resolved by discussion. We assessed agreement using kappa for a core subset of categorisations: agreement was substantial for assessing the adequacy of sequence generation (*k* = 0.63 [95%CI 0.5–0.8]) and allocation concealment methods (*k* = 0.71 [95%CI 0.5–0.8]), and most disagreements concerned conflicting interpretations of whether meagre descriptions should be judged as ‘unclear’ or ‘inadequate’. Agreement was perfect when judging the presence of patient blinding (*k* = 1.00), near-perfect for outcome assessor blinding (*k* = 0.90 [95%CI 0.8–1.0]) and substantial for blinding care-providers (*k* = 0.65 [95%CI 0.3–1.0]); all discrepancies were due to inaccuracies in data extraction and the three disagreements regarding whether care-providers had been masked owed to the misidentification of whether personnel described as blind were treating physicians performing the experimental or comparator test.

We present a descriptive analysis using percentages that reflect the categorical nature of the data. Although we compare frequencies to enhance interpretation, testing for statistical significance was inappropriate as we did not evaluate specific hypotheses.

## Results

### Included trials

The search strategy retrieved 12,892 citations, yielding 103 eligible trials that compared 105 control interventions with 119 experimental interventions. A broad range of test-treatment strategies were evaluated across a wide range of settings. A PRISMA flow diagram and tabulated characteristics of included studies are available elsewhere, along with an analysis of the quality of descriptions of test-treatment interventions [27].

### Outcomes

A total of 149 primary outcomes were reported by 97 of the 103 trials (Table 3). Most studies had a single primary outcome (79/103, 77%), 18 trials measured between 2 and 15 primary outcomes (median 3, IQR: 2–4), and 6 trials failed to clearly specify a primary outcome. Fifty-three trials reported 96 separate measurements of health as a primary outcome, though in 22 of these trials outcomes reflected short-term clinical response or disease status, and not downstream assessments directly measuring the benefits of treatment (Table 3). In 38 trials the primary outcomes were process outcomes, such as diagnostic and treatment decisions, timing, and measures of appropriateness of care.

**Table 3:** Types of outcomes measured as primary endpoints in test-treatment RCTs

Outcome Type	Trials, *n*	(%)	^a^Outcome measurements, *n*	(%)
Patient				
Symptom score	13	(25)	14	(9)
Adverse events	8	(15)	15	(10)
Function	8	(15)	11	(7)
Quality of life	5	(9)	17	(11)
Mortality	4	(8)	4	(3)
Health perception	2	(4)	5	(3)
Psychological morbidity	2	(4)	6	(4)
Absenteeism	1	(2)	1	(1)
Clinical status	9	(17)	9	(6)
Residual disease rate	7	(13)	7	(5)
Recurrent disease rate	6	(11)	7	(5)
Patient outcome total	53	(54)	96	(64)
Process				
Therapeutic yield	17	(45)	20	(13)
Timing of care	8	(21)	8	(5)
Cost	7	(18)	7	(5)
Appropriateness of treatment decision	5	(13)	6	(4)
Diagnostic yield	4	(11)	5	(3)
Process outcome total	38	(39)	46	(31)
Composite outcome				
Adverse patient and process event rate	7	(7)	7	(5)
Primary outcome not defined	6	(6)	0	(0)
Total	103	(100)	149	(100)

^a^Many trials included more than one primary outcome

Half the trials (51%) detailed methods of measurement for the primary outcome with adequate detail to replicate, while neither the measurement method nor timing were described by 17 trials (17%). Incomplete reports most commonly omitted the time at which outcomes were measured (missing for 43/57 partially reported outcomes, 75%). Complete accounts of participant flow, reporting numbers who were eligible, allocated, received interventions, followed-up and analyzed (as recommended by CONSORT [30]) were provided by 44 (43%) trials, of which 20 (19%) also provided a full flow diagram. One study (cluster-randomized) reported none of these details, while the remaining 58 (56%) trials published partial information.

### Risk of bias from randomization, blinding and loss to-follow-up

Methods of sequence generation and allocation concealment were adequate in 59 (57%) and 38 (37%) trials (Table 4). Only 2 and 3 trials described inadequate methods of sequence generation and allocation respectively, the remaining majority provided insufficient descriptions to judge.

**Table 4:** Methodological characteristics of test-treatment trials

Trial Quality Item	Trials, *n*	(%)
Randomized sequence allocation		
Adequate	59	(57)
Inadequate	2	(2)
Unclear	42	(41)
Allocation concealment		
Adequate	38	(37)
Inadequate	3	(3)
Unclear	62	(60)
Blinding		
Patients	5	(5)
Care-providers	4	(4)
Outcome assessors	22	(21)
^a^Single-blind	20	(19)
^b^Double-blind	5	(5)
No blinding	78	(76)
Reporting of primary outcome assessment		
Complete	53	(51)
Partial	33	(32)
Absent	17	(17)
Reporting of participant flow		
Complete	44	(43)
Partial	58	(56)
Absent	1	(1)
Missing data		
Complete	41	(40)
Attrition ≤10%	30	(29)
Attrition >10%	25	(24)
Incomplete, cannot calculate	4	(4)
Unclear if complete	3	(3)
Differential attrition		
≥ 5% between arms	16	(16)
≥ 20% between arms	1	(1)
Intention-to-treat (ITT)		
Patients analyzed as randomized	72	(70)
Complete or imputed data and analyzed as randomized	30	(29)
Not conducted	31	(30)
Inconsistent outcome assessment	21	(20)
Inappropriate subgroup analysis	9	(9)
Sample size		
Power calculation reported	81	(79)
^c^Median trial sample size [IQR]	309	[153–731]
^c^Median study arm sample size [IQR]	166	[72–297]

^a^blinding either patients or care-providers or outcome assessors; ^b^blinding at least two of: patients, care-providers or outcome assessors; ^c^based on numbers randomized in all 103 trials

Few trials reported blinding: 5 (5%) trials reported blinding patients, 4 (4%) blinded care-providers and 22 (21%) blinded outcome assessors. Few trials explicitly stated that blinding was not used. The trials that blinded patients gave both experimental and control tests to all patients (4/5) [32–35] or conducted all tests on samples in a laboratory (1/5) [36] and masked patients from results. Treating clinicians were blinded by being provided with standardized diagnostic reports in both arms [37], by receiving sham diagnostic reports with actual diagnostic decision-making conducted by off-site non-treating clinicians [35], or by non-disclosure of laboratory processes for the length of the trial [36].

Inadequate reporting of primary outcomes made ascertaining the identity of the outcome assessor difficult, hence we often deduced this detail implicitly from the outcome type and descriptions of measurement methods. Blind outcome assessments were achieved using independent expert panels in nine trials (9%), clinicians not involved in patient care in six trials (6%), or by independent research assistants in 7 (7%).

Most trials (60, 58%) used objective primary outcomes (e.g. all-cause mortality, healthcare cost) and standardized measures of health response (e.g. maximal endurance to exercise on the treadmill), and 27% (16/60) of these trials performed blinded evaluations. Subjective primary outcomes were less frequently blinded (7/39, 18%).

**Fig. 2:**
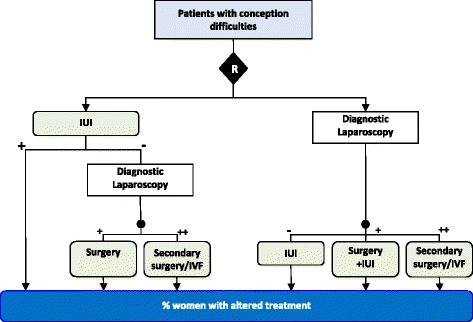
Example of an inappropriate subgroup comparison, leading to differential attrition of >20%. This triage comparison trial compared a strategy of only undertaking diagnostic laparoscopy in women who had failed first-line intrauterine insemination (IUI) rather than undertaking laparoscopy in all women prior to fertility treatment [59]. The primary outcome was the proportion of women experiencing a change in fertility treatment from IUI. The published analysis used the proportion of participants undergoing diagnostic laparoscopy as the denominator rather than the number randomized in each arm. The authors analysis reported a non-significant small increase (experimental 13/23 (56%), control 31/64 (48%); OR = 1.4 [95%CI: 0.5–3.6]). However when the full study population is used a significant *decrease* in the proportion of women receiving a change in treatment is observed (experimental 13/77 (17%), control 31/77 (40%); OR = 0.3 [95%CI: 0.14–0.64]). Excluding participants who did not receive a laparoscopy (70% of experimental group participants, and 17% of comparator arm participants) all experimental group patients who became pregnant during intrauterine insemination treatment were excluded from the effectiveness measurement introducing selection bias

Fifty-nine (57%) studies were incomplete due to the exclusion of participants after randomization (32, 31%) and/or missing outcome data (52, 51%). Missing outcome data were adequately explained in 26 of these trials, 11 provided no description and 25 gave partial accounts that were insufficient to determine the reasons for missing data. The number of participants with missing data on the primary outcome in the 59 trials ranged from 0.1 to 46% of randomized participants (median: 7.0%, IQR: 1.4%–17.6%) and 25 trials excluded more than 10% of the original study population. The proportion of missing data could not be calculated for four trials due to poor reporting. Attrition differed by more than 5% between arms in 21 comparisons made by 16 trials, and in 18 cases (86%) experimental interventions lost the most participants. Attrition differed by more than 20% in only one trial (Fig. 2). Procedures for handling missing data were poorly reported. The majority of trials with missing data performed a complete case analysis (61%, 36/59).

Nine trials (17%) imputed all missing values, while three others imputed partial responses but excluded wholly missing records. No trial reported using multiple imputation methods.

### Risk of bias due to inappropriate handling of primary analyses

Although 72 (70%) of trials analyzed paitents according to their allocated inteventions, the first requirement for an intention-to-treat (ITT) analysis; only 30 (29%) analyzed patients by their assigned groups and had no missing data or imputed missing data, and so comply with the most rigorous definition of intention-to-treat analysis [29]. By comparison 31 trials (30%) failed to analyze patients according to original allocations.

**Fig. 3:**
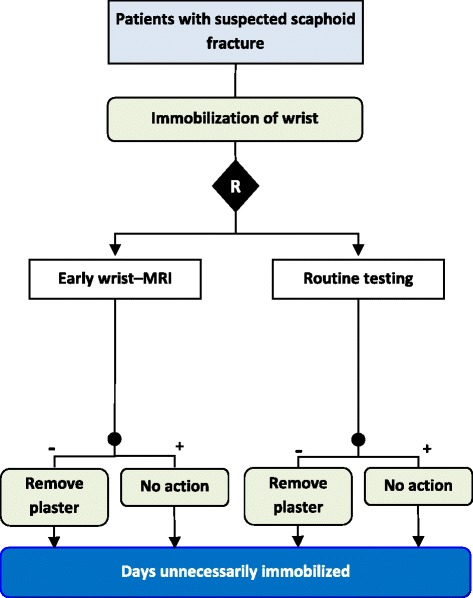
Example of inconsistent measurement of the primary outcome between study arms. The primary outcome of the number of days unnecessarily immobilized is not comparable as the assessment that the plaster is unnecessary and can be removed is determined using MRI findings in the experimental arm and by routine testing (commonly X-ray) in the comparator arm [38]

The majority of trials performed consistent between-arm comparisons (82/103, 80%), either clearly using the same measurement method across all study arms (63, 61%) or assessing test performance outcomes (e.g. diagnostic yield or therapeutic yield–% of patients allocated a particular diagnosis or treatment) for which use of different tests is appropriate (19, 18%). For three trials the outcome was measured in different ways between study arms. For example, a trial of patients with suspected scaphoid fracture compared expedited MRI imaging within 5-days of presentation with standard testing (generally X-rays taking place 2 weeks after immobilization) (Fig. 3). The primary outcome was unnecessary initial immobilization, a treatment decision measure based on observing normal MRI findings in the experimental arm and normal findings on standard imaging in the standard testing arm [38]. In order to achieve true comparability, the same test would have to be used across study arms to determine whether immobilization was truly unnecessary. Consistency could not be determined in the remaining 18 trials (17%) due to lack of reporting.

Comparisons in nine trials (including six with consistent outcome assessments) were judged as unfair due to analysis across improper subgroups; typically this entailed measuring the primary outcome in a single diagnostic or treatment subgroup, such as counting the frequency of antibiotic prescription amongst patients diagnosed with non-pneumonic respiratory tract infection (the study target condition) [39]. Since a patient’s eligibility for contributing to the primary outcome is defined by different tests being evaluated in each arm, these studies are at risk of having produced distorted measures of effect by comparing two groups that are not analogous in patient characteristics. When event rates were recalculated using the more appropriate denominator of all randomized participants, the results of one trial changed direction (Fig. 2).

More than half (60, 58%) the studies were inadequate in either their use of consistent outcome assessments, analysis of patients by allocated group or avoidance of inappropriate exclusions; only 19 trials (18%) performed adequate primary analyses that minimized all assessed risks of selection bias.

### Reporting of power calculations in test-treatment RCTs

Power calculations were reported in 81 (79%) trials. Nearly all related to a specific outcome parameter (79/81, 98%) which matched the primary outcome in 72% (59/81). The remainder either did not define a primary outcome (12, 15%), powered on a single variable when the study evaluated multiple primary outcomes (7, 9%), or used a different variable to power the study (1, 1%).

Study populations ranged from 20 to 5341 participants in individually-randomized trials, and 145 to 972 participants in cluster RCTs. Trials reporting power calculations had considerably larger study samples (median: 408, IQR:157–782) than those omitting this description (median: 212, IQR: 108–304). Trials using patient primary outcomes had slightly larger median study samples (median: 348, IQR: 163–772) compared to those using process outcomes (median 247, IQR: 138–500).

### Risk of type II error in test-treatment RCTs

Of the 79 trials in which a comparison between target and achieved sample sizes could be made (two did not provide power calculation results), 11 (14%) failed to reach 75% of their estimated targets, including four that recruited less than 50%. Of the 13 trials with a ≥5% deficit, 5 (38%) reported difficulties in recruiting, 4 (31%) were stopped early due to either routine uptake of the new test-treat strategy (*n* = 2), or significantly different outcome rates to those expected. None provided details of any stopping rules.

## Discussion

We report on the methodological quality of test-treatment trials unrestricted by clinical setting or publication. Of the 103 test-treatment RCTs we assessed, only two were not found to be at risk of bias using the standard Cochrane criteria. In many instances our appraisals were hindered by insufficient reporting of trial conduct and methods. The choice of primary outcomes raise some concern regarding the usefulness of test-treatment trials, since the majority aimed to answer intermediate questions of process or short-term health impact, whose relevance to long-term health may be questionable [40]. Most trials failed to protect against selection bias by implementing inadequate allocation concealment and/or randomization measures, and this risk was amplified in the two-thirds of trials that performed suboptimal analyses on incomplete populations. The rarity of blinding suggests that approximately 95% of trials produced results that risk reflecting the expectations of participants, clinicians and trialists, particularly the 30% of studies that measured subjective outcomes in unblinded fashion and so were also at high risk of ascertainment bias. These features are all known to cause overestimated treatment effects and potentially spurious findings in trials of treatment interventions [24–26, 41].

### Comparison to existing reviews of trial quality

A previous review of test-treatment RCTs [15] was limited to a sample of 50 published trials ascertained from searches of the top six generalist medical journals (without year restrictions), which reported more favorable frequencies of methodological safeguards than found here (only 10% of our 103 trials were published in these six journals). Blinding of any type was reported in a similar proportion of trials (30% vs 24%), but higher proportions reported allocation concealment (50% vs 37%) and maintained attrition at <10% (80% vs 69%). Differences of this magnitude could occur by chance, but may also reflect better reporting and methodological quality in RCTs published in top generalist medical journals.

Similar reviews have assessed the methodological quality of treatment intervention trials, and we compare our findings for randomization, allocation concealment and blinding with those of an assimilated analysis of 1973 trials of mainly pharmaceutical interventions [26]. In test-treatment trials, methods of sequence generation (57%) and allocation concealment (37%) were more often adequate than in RCTs of treatments (25 and 23%). However, double-blinding was reported in 56% (590/1057) of intervention trials compared with only 5% of test-treatment RCTs (5/103) (based on at least two categories of individual being blinded). Conduct of ITT analyses were rarer amongst test-treatment trials (30%) than treatment trials (48%–57% [42, 43]), and rates of attrition higher, with 25% of our sample excluding >10% of participants compared to only 10% of treatment trials [42, 44]. Lastly, although our review found power calculations were reported *more* frequently by test-treatment trials (79% vs. 27–45% [30, 46]) median sample sizes were somewhat *smaller* than the 425 per arm (IQR: 158–1041) recruited by 225 contemporary (2005–6) parallel-group RCTs [45].

On the other hand, our findings are broadly consistent with appraisals of complex intervention trials; ITT analyses were found in 24% (vs. 30%), although power calculations were reported considerably less often than by test-treatment trials (28% vs 79%) [46]. Rates of blinding are also similar to reviews of surgical trials that reported blinding of patients in 8–15%, care-providers in 0–8% and outcome assessment in 17–35% [47, 48]. Reviews directly comparing non-pharmacologic and pharmaceutical RCTs for osteoarthritis also showed that blinding is significantly less common in complex intervention trials, particularly for patients (24–26% vs. 96–97%) and care-providers (6% vs. 82%) [48, 49].

### Interpretation of findings and implications for practice

The low quality of test-treatment RCTs is partly explained by the suboptimal quality observed across all types of RCT [26], yet the above comparisons indicate that it also reflects methodological challenges that specifically affect test-treatment trials due to the multi-staged nature of their interventions.

As with therapeutic complex intervention trials, the scarcity of blinding in test-treatment RCTs almost certainly reflects the practical and ethical difficulties involved in blinding all trial participants (patients, care-providers and assessors) from multiple elements of lengthy care pathways, which may be invasive and are typified by active patient and clinician participation. For the majority of test-treatment comparisons blinding is likely to be impossible, particularly for clinicians who would need to be masked from the test(s) being used, and possibly also from the test result itself. It is difficult to imagine many clinical situations in which this would be ethical or practicable.

Then again, the degree to which not blinding exposes test-treatment trials to bias cannot necessarily be directly inferred from what we know of treatment trials. Diagnostic tests are decision-making tools, hence it is possible that blinding itself can have unintended consequences on the validity of trial results, for example from removing the clinician’s role from decision-making. In one of our cohort, patients with suspected pulmonary embolism (PE) received treatment directed either by results of a standard ventilation-perfusion (V/Q) scan, or by an initial triage test (‘BIOPED’ combined test: D-dimer blood test, alveolar dead space measurement, and Well’s seven-variable clinical model) followed by V/Q scan if PE could not be ruled out [35]. Interpretation of BIOPED results was passed to non-treating clinicians (trial investigators not involved in patient care) to decide which patients should go on to receive a V/Q scan: BIOPED negative patients (i.e. PE ruled out) received a sham V/Q procedure, while test positives received a true V/Q scan. In order to maintain blinding of clinicians, a fake negative nuclear medicine report was sent to the physicians of patients who had received a sham V/Q scan. By circumventing the contribution of the treating clinician, this study runs the risk of producing treatment effects which could never be reproduced in reality; that is to say its applicability to similar diagnostic settings in practice has been compromised. In short, blinding clinicians is unlikely to prevent performance bias in test-treatment trials, and may even distort the effects we are trying to observe. Blinding of patients and outcome assessments is likely to be particularly important when measuring subjective outcomes that are sensitive to patient and clinician expectations. Research is needed to determine the extent to which this will be feasible, and is important.

We also provide empirical confirmation that test-treatment trials are particularly susceptible to attrition and lack of power. High rates of missing outcome data could indicate that test-treatment RCTs are at an increased risk of losing participants after randomization, perhaps due to the practical difficulties of maintaining patient compliance throughout numerous interventions, longer study periods and potentially more intensive follow-up regimes. The lack of blinding in these trials may also drive drop-out rates and missing responses, since patients dissatisfied or disillusioned with their diagnostic allocations may have been less motivated to comply with the trial’s follow-up protocol. This was documented by authors of one trial evaluating the benefits of investigating patients with suspected fractures using an MRI scan in addition to the usual X-ray, who reported that patients randomized to the MRI arm were more likely to return their questionnaires than those who knew they had not received the new technology [50].

The lower sample sizes observed were unexpected since test-treatment sample sizes generally need to be substantially larger than is usual for treatment trials as health effects are diluted by the subgroup of patients who would receive the same diagnosis and treatment by both test-treat strategies under evaluation [22, 23, 51]. Although we have not considered the validity of the justification of sample sizes in each individual study, the lower than average sample sizes indicate that test-treatment RCTs are likely to be underpowered to detect differences in downstream patient health.

Often an RCT may be used to assess whether there are reductions in the proportions of patients undergoing inappropriate additional investigations or interventions. We observed instances where results were computed based on the numbers undergoing additional investigations or interventions, rather than the total numbers randomized. This introduces bias as those receiving additional testing and treatment are determined post-randomization and are not based on the same information in both study arms [52].

Despite these practical challenges, there is no theoretical reason why test-treatment RCTs cannot minimize the risks of attrition bias and type II error to a similar degree as standard treatment RCTs. Ensuring that published trials are internally valid, however, will require current methods of critical appraisal to be adapted to reflect the particular requirements of test-treatment interventions.

### Strengths and limitations

Our study examines the internal validity of a systematically-derived and unrestricted group of test-treatment RCTs measuring patient outcomes. The cohort comprises a diverse range of test-treatment interventions, conducted across a wide range of clinical settings. We have previously shown [6] that our search was close to complete.

Two factors may have impacted on the reliability of our estimate of trial quality. We only undertook duplicate data extraction for 64% of our sample, however the perfect or near-perfect agreement we found indicates the likelihood of error is small. Lastly, the quality of trial reporting and conduct has improved over the last 15 years [53, 54] so it is possible that trials published since 2007 are of better quality. However, since no guidance on how to resolve the unique issues posed by test-treatment trial designs has been disseminated in the interim, improvements are unlikely to be far-reaching.

## Conclusion and recommendations

There is a clear need to improve the conduct and reporting of test-treatment RCTS, and guidance for trialists to resolve these issues is urgently needed. Existing RCT quality checklists, such as those provided by NICE [55] and Cochrane [31], do not currently address the methodological peculiarities of test-treatment interventions, though could be amended to do so.

Our review emphasizes methodological weaknesses intrinsic to the RCT design when used to evaluate test-treatment interventions. Minimizing attrition poses a much greater practical difficulty for test-treatment trialists than those undertaking standard trials, while blinding and adequate powering present additional challenges which in many circumstances may be impossible to overcome. These pose considerable obstacles to successfully completing test-treatment trials, particularly when viewed alongside evidence that test-treatment interventions are difficult to capture and translate into trial protocols [27], and that they impact on patient health in numerous and highly complicated ways [51]. While the RCT notionally remains the ideal design to evaluate clinical effectiveness, a likely implication of our findings is that it may well have limited success when used to evaluate certain test-treatment interventions.

When an RCT design is used, our findings highlight that standard RCT methods need to be tailored to suit test-treatment interventions. Since blinding clinicians is unlikely to be feasible, the use of more detailed or rigid protocols may serve to limit systematic differences in care provision that occur beyond those generated by using different diagnostic tests for decision-making. Close accounting of clinical behavior, including test use, decision-making and treatment use, will assist investigators to discern between effects due to genuine divergence in test performance from those that reflect artefacts of study design. The noncomparability of outcome measurements between study arms is another unique challenge to test-treatment RCTs that occurs because the intervention we wish to evaluate is itself designed to categorize patients into subgroups. From a clinical perspective it is intuitive to want to know how patients fare between particular treatment categories in order to gauge the appropriateness of decision-making, however the introduction of a third test to ensure comparability of outcomes in all participants may prove practically difficult. Finally, adequate powering requires trialists to inflate estimates based on standard treatment effects by the projected fraction of participants who would receive a difference in diagnosis between test-treatment interventions [51], leading to unattainable patient recruitment targets.

We maintain that protection against bias in RCT studies is not often feasible, and thus these designs should be mandated with care. First, it is essential to examine whether the test-treatment strategies being compared can accommodate the key methodological safeguards, in particular adequate powering, consistent outcome measurement and outcome blinding. In cases where it is impossible to control for bias, smaller scale observational studies and modelling may prove to be more valid. Urgent research is needed to establish: whether blinding is feasible, the impact that not blinding has on the reliability of trial results, and–importantly–the validity of alternative linked-evidence approaches.

## Abbreviations

BIOPED: Bedside Investigation of Pulmonary Embolism Diagnosis
CENTRAL: *Cochrane Central* Register of Controlled Trials
CONSORT: Consolidated Standards of Reporting Trials
EMBASE: Excerpta Medica dataBASE
IQR: Interquartile range
ITT: Intention-to-treat
MEDLINE: Medical Literature Analysis and Retrieval System Online
MRC-CUBE: Medical Research Council carbon-13 urea breath test and eradication
MRI: Magnetic resonance imaging
NICE: National Institute for Health and Care Excellence
PE: Pulmonary embolism
PRISMA: Preferred Reporting Items for Systematic Reviews and Meta-Analyses
RCT: Randomised controlled trial
V/Q: Ventilation-perfusion
